# Age-dependent differences of the depth of olfactory fossa in children

**DOI:** 10.1016/j.bjorl.2021.09.006

**Published:** 2021-11-05

**Authors:** Mehmet Güven, Halil Elden, Atılay Yaylacı, Ebru Mihriban Güven, Ahmet Kara, Ayla Tekin Orha

**Affiliations:** a Sakarya University, Faculty of Medicine, Department of Otorhinolaryngology, Sakarya, Turkey; b Kocaeli University, Faculty of Medicine, Department of Otorhinolaryngology, Kocaeli, Turkey; c Sakarya University, Faculty of Medicine, Department of Anatomy, Sakarya, Turkey; d Kocaeli University, Faculty of Medicine, Department of Anatomy, Kocaeli, Turkey

**Keywords:** Olfactory groove, Multi-slice computed tomography, Keros classification

## Abstract

**Objective:**

With this radio-anatomical study, we aimed to describe the distribution of the depth of the olfactory fossa based on the Keros classification in the pediatric population in our region and to reduce complication rates by providing normative data.

**Methods:**

This was a retrospective study conducted with computed tomography imaging of the paranasal sinuses of 390 pediatric patients referred over a six-year period in Sakarya and Kocaeli University Faculty of Medicine. Patients were divided into 3 groups as 1–6, 6–12, and 12–18 years old. The depth of the olfactory fossa was measured and classified according to the Keros classification. The incidence of Keros asymmetries was also investigated.

**Results:**

The distribution of the depth of a total of 780 olfactory fossa according to the Keros classification was 24.7% Keros I, 65.9% Keros II, and 9.4% Keros III. When the groups were evaluated with each other and within each group, it was seen that the prevalence of Keros I type was significantly higher in the first group (*p* < 0.05), and the prevalence of Keros type II was significantly higher in the second and third groups (*p* < 0.05). Apart from this, the number of Keros type III increased in the third group compared to the first two groups and showed a statistically significant difference (*p* < 0.05). Among all patients, asymmetry of the olfactory fossa was detected in 29 patients (7.4%). Although the number of olfactory fossa asymmetry was low in group I, it was not significantly different between the groups (*p* > 0.05).

**Conclusion:**

In our study, high Keros I rate and low Keros III rate in children aged 1–6 were remarkable. Especially for children under the age of six, questions arise about the validity of the Keros classification. More detailed studies in larger populations, in different ethnicities, and with various age groups are needed.

**Level of evidence:**

Level 3.

## Highlights

• The shape of the anterior skull base changes with age and is directly linked to the development of the paranasal sinuses.
• Considering children under the age of 6, questions arise about the validity of Keros classification.
• The high Keros I ratio and the low Keros III ratio in children aged 1–6 and the high rate of Keros III in the 12–18 age group were remarkable findings.

## Introduction

Today, Endoscopic Sinus Surgery (ESS) in children is the primary treatment option for diseases such as chronic rhinosinusitis, mucocele, nasal polyposis, Cerebrospinal Fistula (CSF), paranasal sinus tumors, and epiphora due to lacrimal duct obstruction.^1^ It is mandatory to define the paranasal sinus anatomical landmarks during the preoperative and intraoperative periods to avoid complications (e.g., intraorbital hematoma with vision loss, CSF leak, and intracranial penetration). Particular attention should be paid to the olfactory fossa and the Anterior Skull Base (ASB) for inadvertent intracranial penetration leading to intracranial injury and CSF leak.^2^ These major complications are associated with surgical manipulation of the ethmoid and frontal sinuses.^3^ According to the literature, these complications are known to occur most frequently in the Lateral Lamella (LL) of the Ethmoid Roof (ER).^4^ The lamina lateralis of the ethmoid bone, which joins laterally with the orbital part of the frontal bone, is one of the most vulnerable points of the ASB.^5^ Therefore, as this lamina deepens, the probability of being traumatized increases in many procedures that may involve the ASB.^6^ It is obvious that better awareness of the anatomy of this region preoperatively and making precise manipulations at this site during the operation will reduce the possibility of complications. Therefore, preoperative paranasal sinus Computed Tomography (CT) imaging, which provides a clear definition of anatomical structures and pathological findings, is the gold standard examination method.^2^, ^7^, ^8^

The ethmoid roof is formed by the cribriform plate medially and is limited by the LL laterally. It represents a continuation of the attachment of the middle turbinate. This anatomic landmark is the thinnest structure of the ER and is therefore termed the “locus minoris resistentiae” in ESS.^2^, ^3^, ^9^, ^10^ The sizes of the ER and cribriform plate can vary considerably.^2^ In the early 1960's, Keros divided the olfactory fossa into three categories according to the length of the LL, and this classification is still used today.^11^ Using a study of 450 adults, he was able to divide the ER configuration into the following three categories: Keros type I: Olfactory fossa is 1–3 mm deep, and LL across the sulci is short; Keros type II: Olfactory fossa is 3–7 mm deep, and the LL constitutes a large portion of the roof; Keros type III: Olfactory fossa is 8–16 mm deep, and it is called a “dangerous ethmoid” by Kainz and Stamberger.^12^ In Keros type III, the ER lies at a significant level above the cribriform plate. In this type, the thin LL forms a larger ER component, which is not protected by the thick frontal bone. Therefore, the Keros type III is the most vulnerable type and is considered to pose a significant risk for ESS.^7^, ^8^

Although ESS has also recently become the standard therapy for chronic sinus disease and its complications in childhood,^13^, ^14^, ^15^ there is little literature on the developmental changes in the ethmoid roof and the corresponding position of the lamina cribrosa. However, it is clear that the PS of children differs in size and shape from those of adults. Their development is directly influenced by the development of the skull base and the dentition.^15^, ^16^

In studies conducted to date, it has been determined that the depth of the olfactory fossa varies in adult age groups. However, there has not been any detailed study conducted on the depth of the Olfactory Fossa (DOF) according to age in childhood. This radio-anatomical study aimed to determine the DOF according to age of the population under 18 years old in our region and to reduce the complication rates by providing normative data.

## Methods

The research protocol was submitted to and approved by the local ethics committee (nº 71522473/050.01.04/272) and was conducted in accordance with the ethical regulations of the Declaration of Helsinki and in adherence to the laws and regulations. Paranasal sinus CT images of a pediatric population 1–18 years old at Sakarya and Kocaeli University Faculty of Medicine between January 2013 and October 2019 were analyzed. All images were created using a 64-detector CT device (Toshiba Aquilion 64MDCT, Toshiba Medical Systems, Otawara, Japan) with a slice range up to 2-mm. A history of surgery or trauma in the paranasal sinuses, malignant disease of paranasal sinuses, the presence of diffuse nasal polyposis, cystic fibrosis, or Kartagener’s syndrome, which could change the anatomy of the paranasal sinuses and skull base, the presence of congenital facial anomaly, inappropriate CT images and children under the age of 1 were determined as exclusion criteria. All eligible CT images between the ages of 1–18, outside the exclusion criteria, were included in the study.

**Figure 1:**
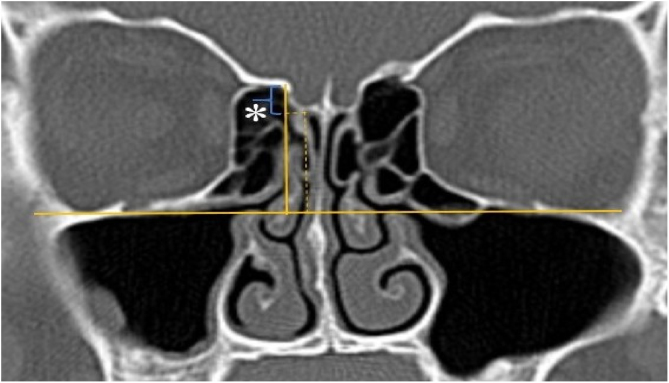
Measurement of olfactory fossa depth (*).

**Figure 2:**
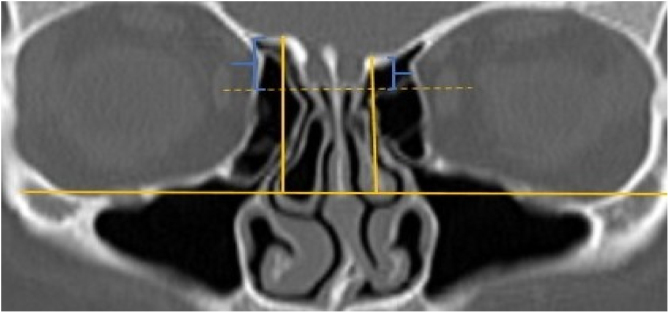
Asymmetry of olfactory fossa depth.

**Figure 3:**
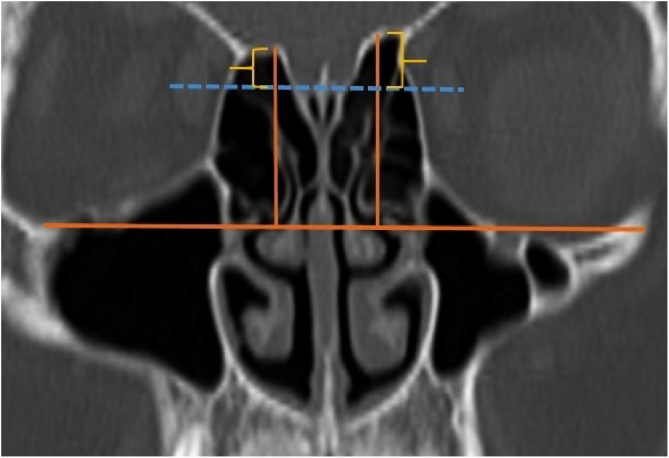
Another example of asymmetry of olfactory fossa depth.

A total of 390 CT studies belonging to the pediatric population that met the inclusion criteria were included in the study. Patients were divided into 3 groups according to their age: group I (ages between 1–6, n = 56), group II (ages between 6–12, n = 176), and group III (ages between 12–18, n = 158). Paranasal sinus CT images of the study groups were analyzed in the coronal plane with the DOF and asymmetry. The horizontal line joining both infraorbital nerves was taken as the base to ensure standardization in measurement of the DOF; from here, a perpendicular line was drawn to the medial border of the fovea ethmoidal is, then a perpendicular line was drawn to the most lateral part of the cribriform plate. The difference between the lengths of these two perpendicular lines was noted as the DOF. Measurements were then grouped according to the Keros classification (Fig. 1). In addition to this, the length difference between the right and left olfactory fossa depths was also noted as Keros asymmetry (Figure 2, Figure 3).

### Statistical analysis

Statistical analyses were performed using IBM SPSS Statistics 22 commercial software (SPSS Inc. An IBM Co. Armonk, NY). Kolmogorov-Smirnov test was used to evaluate the normality of the distribution of variables and based on this we chose parametric or non-parametric statistics. Kruskal-Wallis test was used to compare continuous variables and whether there were differences between measures. Tamhane’s post-hoc test was used to locate eventual differences. Continuous variables are presented as mean ± standard deviation if they showed normal distribution and as median [interquartile range] value if they did not show normal distribution. Categorical variables were compared using Chi-Square test and are presented as number (n) and percentage (%). Chi-Square tables were evaluated by post-hoc test. A *p*-value of <0.05 was considered significant.

## Results

Paranasal Sinus CT scans of a total of 390 patients were analyzed. The mean age of the patients was 4.84 ± 1.41 in group I, 9.86 ± 1.45 in group II, and 15.29 ± 1.61 in group III. For the distribution of the 390 patients, which included 189 females and 201 males, by groups: in the group I, 44.6% (n = 25) female and 55.4% (n = 31) male; in group II 46.6% (n = 82) female, 53.4% (n = 94) male, and in group III 51.9% (n = 82) female and 48.1% (n = 76) male. There was no statistically significant difference in the gender distribution between the groups (*p* > 0.05) (Table 1).

**Table 1:** An overview of the distribution of the groups according to gender, depth of the olfactory fossa and Keros classification.

		Total	Group I	Group II	Group III	*p*
			(1–6 years)	(6–12 years)	(12–18 years)	
**Gender**	Female	189 (48.5%)	25 (44.6%)	82 (46.6%)	82 (51.9%)	0.517
	Male	201 (51.5%)	31 (55.4%)	94 (53.4%)	76 (48.1%)	
**Depth of the olfactory fossa**^a^			3.72 [1.67]	4.8 [1.7]	5.6 [2.01]	<0.001
**Keros classification**	Keros 1	193 (24.7%)	60 (53.6%)	85 (24.1%)	48 (15.2%)	<0.001
	Keros 2	514 (65.9%)	49 (43.8%)	252 (71.6%)	213 (67.4%)	
	Keros 3	73 (9.4%)	3 (2.7%)	15 (4.3%)	55 (17.4%)	

a Median (Interquartile Range) mm.

The parameters on the left and right sides were regarded as separate cases for further analysis, therefore 780 cases in 390 pediatric patients were assessed. The Keros distribution of the DOF’s of these 780 cases were 24.7% Keros I, 65.9% Keros II, and 9.4% Keros III of the total of 780 olfactory fossa depths analyzed between 1–18 years of age. 53.6% Keros I, 43.8% Keros II and 2.7% Keros III were observed in group I. In group II, Keros I rate was 24.1%, Keros II rate was 71.6%, and Keros III rate was 4.3%. In group III, Keros I rate was 15.2%, Keros II rate was 67.4%, and Keros III rate was 17.4% (Table 1). When the groups were evaluated with each other and within each group, it was found that the Keros I rate was significantly higher for group I (*p* < 0.05) and the number of Keros II was significantly higher in group II and group III (*p* < 0.05). In addition, the Keros III rate was found to be higher in group III than in the first two groups, which showed a statistically significant difference (*p* < 0.05).

The median value of the DOF was 3.72 [1.67] mm in the first group, 4.80 [1.70] mm in the second group, and 5.60 (2.01) mm in the third group. When the DOF values were examined between the groups, there was a statistically significant difference for all three groups (*p* < 0.05) (Table 1).

Olfactory fossa asymmetry was detected in 29 patients (7.4%) in total. Olfactory fossa asymmetry was detected in 1 patient (1.8%) in group I, 14 patients (8%) in group II, and 14 patients (8.9%) in group III. When the length differences between the right and left olfactory fossa asymmetry in millimeters were evaluated, the median values of group I, II, and III were 0.28 [0.27] mm, 0.40 [0.50] mm, and 0.41 [0.60] mm, respectively. Although the number of olfactory fossa asymmetries was lowest in group I, there was no significant difference between the groups (*p* > 0.05).

## Discussion

The shape of the ASB changes with age and is directly linked to the development of the paranasal sinuses.^15^, ^16^ The ethmoid sinuses are already established at the time of birth and grow in size in all directions with increasing age.^15^, ^16^, ^17^ Ethmoid air cells continue to grow until late puberty or until they reach compact bone.^18^ This development affects the shape of the olfactory fossa and is held to be responsible for the different appearances of the ethmoid roof.^15^, ^19^ In light of this information, the aim of the present study was to analyze the DOF in children according to age.

Today, there is a consensus on the DOF in adult patients. However, very few studies have been conducted on this parameter in the pediatric age group. Anderhuber et al. studied children aged between zero and 14-years. They detected Keros Type I in all children aged 0–1. In addition to this, in the patients aged 1–14 years of age: 14.2% of cases had Keros type I, 70.6% had type II, and the rest had Keros type III. The 1–14 age group results were reported to be similar to the adult group.^15^ Our results of patients aged between 1–18 years with 780 olfactory fossa anatomy analysis showed that 24.7% of the cases had Keros type I, 65.9% of them had Keros type II, and 9.4% of them had Keros type III. Although the age scale of these results is different, it is seen that they are compatible with the results of Anderhuber et al.^15^ Başak et al. reported these frequencies as 9% for type I, 53% for type II, and 38% for type III for children between the ages of 8 and 18 years old.^7^ Güldner et al. found 28% of cases with type I, 51% type II, and 21% type III with a childhood patient group aged 5–15 years old.^2^ In our study, children aged 1–18 years old participated in the study. The frequency differences between our study and the other three studies conducted in the pediatric age group can be explained by these age differences (Table 2).

**Table 2:** An overview of the distribution of the different Keros types in pediatric groups according to the literature.

Reference	n	Modality	Type I	Type II	Type III
Pediatric groups					
Anderhuber et al.	272	CT	14%	71%	15%
Basak et al.	64	CT	9%	53%	38%
Güldner et al.	116	CBCT	28%	51%	21%
Present Study	390	CT	24.7%	65.9%	9.4%

CT, Computed Tomography; CBCT, Cone Beam Computed Tomography.

The evaluation of DOF according to age in our study showed that Keros distributions in group I were 53.6% Keros I, 43.8% Keros II, and 2.7% Keros III. No study was found in the literature other than the study of Anderhuber et al.^15^ investigating DOF for this age range. However, in the study of Anderhuber et al.^15^ this age range was not given as a separate group. They detected Keros type I in all children aged 0–1. Interestingly, in our study Keros I was much more frequent in group I than in group II and group III (53.6% vs. 24.1% and 15.2%). In addition, the high Keros I rate, and the low Keros III rate were noteworthy in children aged 1–6 years (Group I). These results can be explained by the continued development of the ASB and olfactory fossa in this age group. In children under six years of age, bony structures are such that they are more fragile,^7^ and thus the risk of complication increases. It is necessary to be more careful when performing ESS in this age group.

Güldner et al.^2^ suggested that their results, which differ from the studies in the literature, can be explained by the development of the ethmoid roof during childhood. In our study, we analyzed the childhood age patients by dividing them into 3 groups as 1–6 years, 6–12 years, and 12–18 years old. Since there is no ASB in children younger than 1-year, as previously reported in the literature, we have not included them in our study.^18^ Our study is the only study that classifies the DOF according to age with the Keros classification in children. Our results showed that the Keros I rate was significantly higher in group I than the other two groups. Also, it was observed that the rate of Keros II was significantly higher in the second and third groups (*p* < 0.05). Furthermore, the rate of Keros III was found to be increased in the third group (aged 12–18) compared to the first two groups and this showed a statistically significant difference (*p* < 0.05). Possible explanations for these findings might be the development of the ethmoid roof growth until adulthood. There are questions about the validity of the Keros classification in childhood, especially under the age of six.

When studies on Keros asymmetry in adult patients are examined, there are articles in which 59% of Keros asymmetry is detected, but there are also studies that find this rate as 9.5%, 10%, and 12% with similar methods.^3^, ^8^, ^20^, ^21^ Another study in which children aged 7–18 were included, the Keros asymmetry rate was found to be 15% for the pediatric group.^22^ We found no other study in the literature than our study regarding Keros asymmetry in children. In our current study, the rate of Keros asymmetry was found to be 1.8% in group I, 8% in group II, and 8.9% in group III. Based on these findings, we think that surgeons dealing with pediatric ESS should be knowledgeable and careful about this difference in order to reduce the complication rates associated with asymmetric olfactory fossa anatomy.

In this study, we obtained normative data on the depth of the olfactory fossa in children according to age. In our study, the high Keros I rate and the low Keros III rate in children aged 1–6, and the high rate of Keros III in the 12–18 age group were remarkable findings. Considering children under the age of 6, questions arise about the validity of the Keros classification. More detailed studies on a larger pediatric population are needed on this subject.

## Compliance with ethical standards

All procedures performed in studies involving human participants were in accordance with the ethical standards of national research committee and with the 1964 Helsinki declaration and its later amendments or comparable ethical standards.

The research protocol was submitted to and approved by the Sakarya University Ethics Committee (02/12/2019-387).

## Conflicts of interest

The authors declare no conflicts of interest.
